# Psychometric properties of the perceived stress scale (PSS‐10) among pregnant women in China

**DOI:** 10.3389/fpsyt.2024.1493341

**Published:** 2024-12-24

**Authors:** ZiYang Zhang, Qingzhi Wang

**Affiliations:** ^1^ St. Luke’s College of Nursing, Trinity University of Asia, Quezon City, Philippines; ^2^ Department of Community and Health Education, School of Public Health, Xuzhou Medical University, Xuzhou, China; ^3^ Center for Medical Statistics and Data Analysis, Xuzhou Medical University, Xuzhou, China

**Keywords:** perceived stress, pregnant women, psychometric properties, exploratory factor analysis, confirmatory factor analysis, China

## Abstract

**Background:**

Pregnancy, a transformative phase, is often fraught with considerable psychological stress. Within the context of Chinese culture, characterized by intricate family dynamics, societal expectations, and deeply rooted traditional beliefs, the manifestation of stress during pregnancy may present with distinct nuances. The adaptation and validation of the Perceived Stress Scale (PSS-10) for the Chinese context are pivotal for a nuanced understanding and effective intervention for the stressors encountered by pregnant women in China.

**Methods:**

This study enrolled a cohort of 990 pregnant women who completed both the PSS-10 and the Chinese Mental Health Scale (CMHS). The internal consistency reliability was assessed using Cronbach’s α and McDonald’s omega. The construct validity was explored through Exploratory Factor Analysis (EFA), while Confirmatory Factor Analysis (CFA) was utilized to validate the scale’s structural integrity. Criterion-related validity was established by correlating PSS-10 scores with CMHS scores, thereby assessing the scale’s convergent and discriminant validity.

**Results:**

The result demonstrated PSS-10 had robust internal consistency, with Cronbach’s α coefficients and McDonald’s omega (Composite reliability) were more than 0.70 for the scale and its constituent sub-factors. EFA and parallel analysis revealed two salient factors with eigenvalues surpassing 1.0, which accounted for 60.58% and 63.22% of the variance among the second and third trimester samples, respectively. The CFA confirmed the two-factor model’s congruence with the PSS-10’s structure in both sub-samples, with excellent model fit indicated by the fit indices: Standardized Root Mean Residual (SRMR) below 0.08 and both Comparative Fit Index (CFI) and Goodness of Fit Index (GFI) above 0.90. Additionally, the correlation analysis with CMHS scores substantiated the PSS-10’s concurrent validity.

**Conclusion:**

The PSS-10 exhibits commendable psychometric properties, rendering it a pertinent and reliable instrument for assessing perceived stress among pregnant women in China. This validation underscores the PSS-10’s utility in psychological research and clinical practice pertaining to Chinese pregnant populations.

## 1 Introduction

Pregnancy is a natural biological state, an important life event for women, and also a strong psychological stress process (1). Pregnant women need to adapt to physiological reactions such as morning sickness and decreased appetite, as well as psychological pressures such as worrying about the health of the fetus and parenting methods (2). Pregnancy stress is a state of physical and mental imbalance caused by the mismatch between the various needs of pregnant women and their physiological and psychological responses during pregnancy (3). Maternal stress during pregnancy arises from a complex interplay of individual, social, and physical factors, posing risks to both the mother and fetus (4). Physical conditions such as hypertension, gestational diabetes, persistent nausea, and poor sleep quality are commonly associated with increased stress, compounded by frequent visits to obstetrics and gynecology clinics (5). On an individual level, stress often stems from a lack of knowledge about pregnancy, difficulties in adapting to the maternal role, and uncertainties about childbirth and parenting (6, 7). Social and environmental factors, including insufficient family support, financial strain, limited access to quality healthcare, and cultural expectations, further amplify stress (7, 8). Pregnant women in challenging circumstances, such as single mothers, those working while pregnant, or those in resource-poor settings, face heightened psychological pressures (6). Variations in social support systems and family structures across cultural contexts also influence stress levels, manifesting in symptoms like fear, anxiety, low mood, and irritability (3, 9). These stressors, if unaddressed, threaten maternal mental health and contribute to adverse outcomes such as preterm birth, low birth weight, and miscarriage, emphasizing the need for comprehensive care and support systems globally.

Stress is a common concern during pregnancy, with studies indicating that a significant proportion of pregnant women, ranging from 6.0% to 16.7%, report experiencing high levels of stress, and the prevalence of mild to moderate stress is even more widespread, affecting between 13.6% and as much as 91.86% of expectant mothers (10–12). During pregnancy, women undergo physiological changes such as hormonal fluctuations and also face changes in their social, life, family, and even work environments, which can lead to psychological stress responses (13, 14). An increasing body of research indicates that maternal stress during pregnancy can lead to increased levels of corticotropin-releasing hormone (CRH), adrenocorticotropic hormone (ACTH), and cortisol in the bloodstream (14, 15). These pregnancy stress hormones have the potential to cross the placenta, influencing the development of the fetal hypothalamic-pituitary-adrenal (HPA) axis, limbic system, and prefrontal cortex (16). Such biological changes may result in adverse pregnancy outcomes such as miscarriage, preterm birth, low birth weight, and postpartum depression. Considering the high incidence of pregnancy-related stress and its significant impact on the health of both mother and child, it is imperative to identify stress early in pregnancy using validated and reliable assessment methods (17, 18).

The Perceived Stress Scale (PSS) is a widely recognized psychological tool for measuring perceived stress levels over the past month, focusing on the general experience of unpredictability, uncontrollability, and overload rather than specific events (19). Originally with 14 items, the PSS was streamlined to 10 items to enhance its reliability (20). The PSS-10 has been validated for its test-retest reliability, Cronbach’s alpha, and factorial validity in diverse populations and languages (21–23). Despite its widespread use, ongoing psychometric evaluation is essential to ensure its applicability in various cultural and demographic contexts.

In China, the PSS-10 was adapted into Simplified Chinese in 2003 (24), and has since been validated in specific populations such as adolescents (25), nurses (26), and community groups (27). While these studies confirm the scale’s utility in these groups, its applicability to pregnant women remains unexplored. Pregnant women represent a unique population for stress assessment due to the distinct physiological and psychological changes they undergo, including hormonal fluctuations, heightened emotional sensitivity, and specific stressors related to childbirth, fetal health, and maternal role adaptation (4). These factors, combined with cultural and societal expectations surrounding pregnancy in China, create a unique stress profile that requires targeted validation of the PSS-10. This population presents a valuable opportunity to expand the psychometric evidence for the PSS-10. Validating the scale in Chinese pregnant women can enhance its utility in capturing stress levels during a critical life stage, addressing a significant gap in the literature. Moreover, it provides a framework for tailoring interventions to manage maternal stress and contributes to a broader understanding of how cultural and physiological factors interact in the context of perceived stress, enriching the global applicability of the measure.

This study aimed to evaluate the psychometric properties of the PSS-10 in a sample of Chinese pregnant women. To achieve this, the sample was divided into two groups: one for exploratory factor analysis (EFA) to identify the latent factor structure of the PSS-10 and the other for confirmatory factor analysis (CFA) to validate the identified structure. The specific objectives of the study were to: (a) identify the underlying factor structure of the PSS-10 using EFA; (b) assess the reliability of the PSS-10 through Cronbach’s α and McDonald’s omega analyses; (c) use CFA to validate the factor structure identified by EFA and assess the structural validity of the PSS-10 within the target population; (d) perform multiple-group CFA to examine its measurement invariance and determine whether the same underlying constructs are consistently captured across samples from the second and third trimesters; and (e) test its concurrent validity by evaluating its ability to predict maternal mental health outcomes, as measured by the validated Chinese Mental Health Scale (28).

## 2 Methods

### 2.1 Study design

This research was conducted within the framework of a validation study, which is a distinct study design that differs fundamentally from a traditional cross-sectional survey. Validation studies follow specific protocols aimed at assessing the accuracy and reliability of a measurement tool, rather than merely describing population characteristics or testing hypotheses. In line with advancements in psychometric research, it has been recognized that the principles underpinning cross-sectional designs are not entirely aligned with the methodological requirements of validation research (29, 30). For this study, the design was meticulously tailored to adhere to established validation protocols to evaluate the psychometric properties of the PSS-10 scale among pregnant women. The research involved multiple stages, including planning, determining an appropriate population and sample size, data collection, and conducting rigorous psychometric analyses based on scientifically recognized standards. These steps ensured the robustness of the validation process and the reliability of the findings regarding the scale’s ability to measure perceived stress in this specific population. Ethical approval for this study was obtained from the Institutional Review Board (IRB) of Xuzhou Medical University and the Huai’an Maternal and Child Health Hospital. The procedures used in this study in accordance with the Declaration of Helsinki. All participants were aware of the stakes involved in participating in the study and gave written informed consent.

### 2.2 Participants

The participants in this study were first-time pregnant women (primiparas) who were in the second and third trimester of pregnancy and received obstetric outpatient services at Huai’an Maternal and child health hospital during the period from July to December 2023. Inclusion criteria: Primiparas who underwent antenatal care in our hospital during the study period were willing to participate in this study. Exclusion criteria were: (1) women with mental disorders, (2) women with intellectual impairment, and (3) women who refused to participate in the survey. The sample size was determined based on recommendations from the literature, which suggest a minimum of 10-30 participants per observed variable for CFA and a minimum total of 300 participants for SEM (31).

### 2.3 Instruments

#### 2.3.1 Demographic characteristics

Basic demographic attributes encompass a spectrum of personal and familial factors, including but not limited to: age, education level, occupation, monthly family income, residence, family structure, gynecological disease, miscarriage history, marital relationships, parents in-law relationships.

#### 2.3.2 The perceived stress scale

The Perceived Stress Scale (PSS-10) is a psychometric instrument designed to quantify an individual’s perception of stress (19, 32). It comprises a unidimensional set of ten items, each accompanied by a Likert-type response scale ranging from 0 to 4, thereby yielding a total score that spans the interval from 0 to 40. The ordinal scoring system is structured such that an ascending score denotes an increment in the level of perceived stress experienced by the respondent. The PSS-10 is underpinned by a two-factor model, where the items are bifurcated into forward- and reverse-scored components. Specifically, six items (labeled 1, 2, 3, 6, 9, and 10) are categorized as forward-scoring, aligning such that higher values on these items reflect increased stress. Conversely, the remaining quartet of items (4, 5, 7, and 8) are reverse-scored, indicating that higher scores on these items are indicative of a diminished perception of stress relative to the forward-scoring items.

#### 2.3.3 The Chinese mental health scale

The Chinese Mental Health Scale (CMHS) is a widely recognized and extensively utilized assessment instrument for mental health screening within the Chinese population (28). The CMHS is structured around ten distinct subscales designed to evaluate various dimensions of psychological well-being and distress: (1) interpersonal tension; (2) poor psychological endurance; (3) poor adaptability; (4) psychological imbalance; (5) emotional disorder; (6) anxiety; (7) depression; (8) hostility; (9) stubbornly biased; and (10) somatization. Each of the ten subscales is comprised of eight individual items, resulting in a total of 80 items for the comprehensive assessment. Participants are required to rate each item on a 5-point Likert scale, with anchors ranging from 1 (not at all) to 5 (nearly every day). The aggregate score of the CMHS is determined by summing the scores across all items and dividing by the total number of items (80), yielding a total average score that serves as an index of overall mental health status. Individual subscale scores provide insight into the presence and severity of issues within the specific domains of mental health. The CMHS scale achieved an excellent Cronbach’s alpha of 0.97 in this study, reflecting a strong internal consistency.

### 2.4 Data collection

For the execution of our study, a cohort of nurses from maternity clinics was meticulously selected and appointed as investigators. These nurses underwent a comprehensive training program, specifically designed to equip them with the requisite skills for data collection, in a concentrated one-day session. Upon encounter, pregnant women fulfilling these inclusion and exclusion criteria, while visiting an obstetrical clinic, were approached by the investigators. The investigators were tasked with elucidating the implications, benefits, and requirements of study participation to potential participants. Following the dissemination of comprehensive information, and upon securing the voluntary affirmation of consent through signature on the informed consent form, the pregnant women were requested to complete the study questionnaire immediately on-site. This process ensured that the collection of data was performed in a standardized and controlled environment. Subsequent to questionnaire completion, the investigators performed an immediate review, identifying and soliciting the resolution of any missing data points on the spot. This approach was instrumental in upholding the integrity and completeness of the collected data. To further ensure the quality and reliability of the data, the investigative team was subject to a rigorous oversight regime. Supervisors implemented a weekly monitoring schedule and convened regular quality control meetings. These sessions were designed to proactively identify and address any issues that emerged during the data collection process, thereby providing formative feedback and guidance to the investigative team. The outcome of this meticulous process was the distribution of 1000 questionnaires, yielding 990 fully completed and valid responses, culminating in a recovery rate of 99.0%.

### 2.5 Statistical analysis

The preliminary phase of the analysis was dedicated to the computation of descriptive statistics for the characteristics of the samples. A comparative demographic assessment between the two samples was executed utilizing independent samples t-tests for continuous variables and chi-square tests for categorical variables, thereby facilitating an examination of demographic variable discrepancies. Subsequently, an itemized descriptive analysis of the Perceived Stress Scale (PSS-10) was undertaken, encompassing the calculation of the mean and standard deviation (SD) for each item.

The structural validity of the PSS-10 was examined by exploratory factor analysis (EFA) and confirmatory factor analysis (CFA). The total sample samples from the second and third trimesters was randomly and equally divided into group 1 for EFA to build the model and group 2 for CFA to verify the model, respectively. The Kaiser–Meyer–Olkin (KMO) measure, with a recommended threshold of KMO > 0.6 (33), and Bartlett’s Test of Sphericity was employed to confirm whether our data were suitable for factor analysis (34). In EFA, the extraction of factors was accomplished via maximum likelihood, followed by the application of the Varimax orthogonal rotation method. Factors were extracted based on two criteria: (1) factors with eigenvalues greater than 1 and (2) items with factor loadings greater than 0.40 (35). Meanwhile, we conducted a parallel analysis, a data-driven approach to determine the number of factors. Specifically, the eigenvalues from EFA were compared with the random values generated by the Monte Carlo method. We performed 1000 simulations with a threshold of 0.95 for the effective eigenvalues.

The internal reliability of the PSS-10 was assessed using Cronbach’s alpha (α), the most commonly used index for evaluating internal reliability According to established classifications, α values were interpreted as follows: ≥ 0.9 indicating excellent reliability, 0.7 ≤ α < 0.9 as good, and 0.6 ≤ α < 0.7 as acceptable (36, 37). In addition, McDonald’s omega (composite reliability) was calculated as a complementary measure of reliability, with a threshold of acceptability set at ≥ 0.7 (38). This dual approach provides a more robust evaluation of the scale’s internal consistency, addressing potential biases associated with relying solely on Cronbach’s α.

The theoretical model identified in EFA was further tested by CFA using the second sample. In CFA, employing maximum likelihood estimation to appraise the congruence of the model with the empirical data. The dual-factor model of the PSS-10 was subjected to scrutiny at the second and third trimesters of pregnancy, with the objective of evaluating the adequacy of the factor structure. A constellation of indicators was utilized to assess model fit, including the chi-square to degrees of freedom ratio (χ2/df), Goodness of Fit Index (GFI), Comparative Fit Index (CFI), Tucker-Lewis Index (TLI), Root Mean Square Residual (RMR), and Root Mean Square Error of Approximation (RMSEA). A model was considered to demonstrate satisfactory fit if the indices met the following criteria: CFI, GFI, and TLI values ≥ 0.95 indicated a good fit, while values between 0.90 and < 0.95 suggested an acceptable fit; RMSEA and RMR values close to or below 0.06 signified a good fit, whereas values below 0.08 indicated adequate fit (39, 40).

Furthermore, the measurement invariance of the PSS-10 across the second and third trimesters of pregnancy was scrutinized through a sequence of hierarchical models: configural, metric, scalar, and strict. The configural model ascertained the invariance of the PSS-10’s two-factor structure across groups, while the metric model-imposed equality constraints on factor loadings. The scalar model extended these constraints to include intercepts, and the strict model further imposed uniformity on factor loadings, intercepts, and error variances.

In the final phase of analysis, Pearson correlation coefficients were calculated between PSS-10 scores and the diverse construct scores of the Chinese Mental Health Scale (CMHS), thereby assessing the concurrent validity of the PSS-10 scores.

All analytical procedures were conducted utilizing SPSS 26.0 (IBM, Armonk, NY, USA), R4.2.2 (R Core Team, Vienna, Austria) and AMOS 21.0 statistical software (IBM, Armonk, NY, USA). Statistical significance was determined with a two-tailed p-value threshold of less than 0.05.

## 3 Results

### 3.1 Sociodemographic characteristics of the sample

The descriptive statistics of the study sample are delineated in **Table 1**, encapsulating a cohort of 990 pregnant women. This sample was stratified into two sub-samples based on gestational age: 403 participants were in their second trimester, while 587 were in the third trimester. The demographic composition of the sample revealed that the majority of the participants, specifically 68.08%, fell within the age bracket of 25 to 34 years. A significant proportion, amounting to 83.84%, had attained an educational level exceeding nine years of formal schooling. Furthermore, a substantial majority, representing 70.81% of the sample, were residents of urban locales. Notably, an analysis of the educational attainment among the pregnant women in the second trimester disclosed a statistically significant higher level of education when juxtaposed with their counterparts in the third trimester (χ2 = 16.526, P<0.001). Additionally, the proportion of second-trimester participants residing in rural settings was found to be significantly higher than that of those in the third trimester (χ2 = 14.372, P<0.001). There were no statistically significant disparities between the two sub-samples concerning other sociodemographic attributes.

**Table 1:** Sociodemographic characteristics of the sample (N=990).

Variable	Overall (n=990)	Second trimester (n=403)	Third trimester (n=587)	χ^2^	Cramér’s V/Yule’s Phi	p-value
Age				χ^2(2)^ =1.436	0.038	0.488
17-24	200 (20.20%)	76 (18.86%)	124 (21.12%)			
25-34	674 (68.08%)	283 (70.22%)	391 (66.61%)			
35-42	116 (11.72%)	44 (10.92%)	72 (12.27%)			
Education level				χ^2(1)^ =16.526	-0.129	<0.001
Below 9 Year Education	160 (16.16%)	42 (10.42%)	118 (20.10%)			
Above 9 Year Education	830 (83.84%)	361 (89.58%)	469 (79.90%)			
Occupation				χ^2(2)^ =1.625	0.041	0.444
Workless	361 (36.46%)	140 (34.74%)	221 (37.65%)			
Physical labor	338 (34.14%)	136 (33.75%)	202 (34.41%)			
Mental labor	291 (29.39%)	127 (31.51%)	164 (27.94%)			
Per capita monthly household income				χ^2(3)^ =7.286	0.086	0.063
Less than 3000 RMB	82 (8.28%)	28 (6.95%)	54 (9.20%)			
3000-5000RMB	291 (29.39%)	108 (26.80%)	183 (31.18%)			
5000-7000RMB	244 (24.65%)	96 (23.82%)	148 (25.21%)			
More than 7000RMB	373 (37.68%)	171 (42.43%)	202 (34.41%)			
Residence				χ^2(1)^ =14.372	-0.121	<0.001
Rural	289 (29.19%)	91 (22.58%)	198 (33.73%)			
Urban	701 (70.81%)	312 (77.42%)	389 (66.27%)			
Family structure				χ^2(2)^ =0.273	0.017	0.872
Nuclear Family	469 (47.37%)	191 (47.39%)	278 (47.36%)			
Extended Family	490 (49.49%)	198 (49.13%)	292 (49.74%)			
Joint Family	31 (3.13%)	14 (3.47%)	17 (2.90%)			
Gynecological disease				χ^2(1)^ =0.426	0.021	0.514
No	821 (82.93%)	338 (83.87%)	483 (82.28%)			
Yes	169 (17.07%)	65 (16.13%)	104 (17.72%)			
Miscarriage history				χ^2(1)^ =0.460	-0.022	0.498
No	629 (63.54%)	251 (62.28%)	378 (64.40%)			
Yes	361 (36.46%)	152 (37.72%)	209 (35.60%)			
Marital relationships				χ^2(1)^ =2.995	-0.055	0.084
Unsatisfied	71 (7.17%)	22 (5.46%)	49 (8.35%)			
Satisfied	919 (92.83%)	381 (94.54%)	538 (91.65%)			
Parents in-law relationships				χ^2(1)^ =0.007	0.003	0.936
Unsatisfied	161 (16.26%)	66 (16.38%)	95 (16.18%)			
Satisfied	829 (83.74%)	337 (83.62%)	492 (83.82%)			

The subscript numbers in parentheses represent the chi-square test degrees of freedom (df).

### 3.2 Exploratory factor analysis

**Figure 1:**
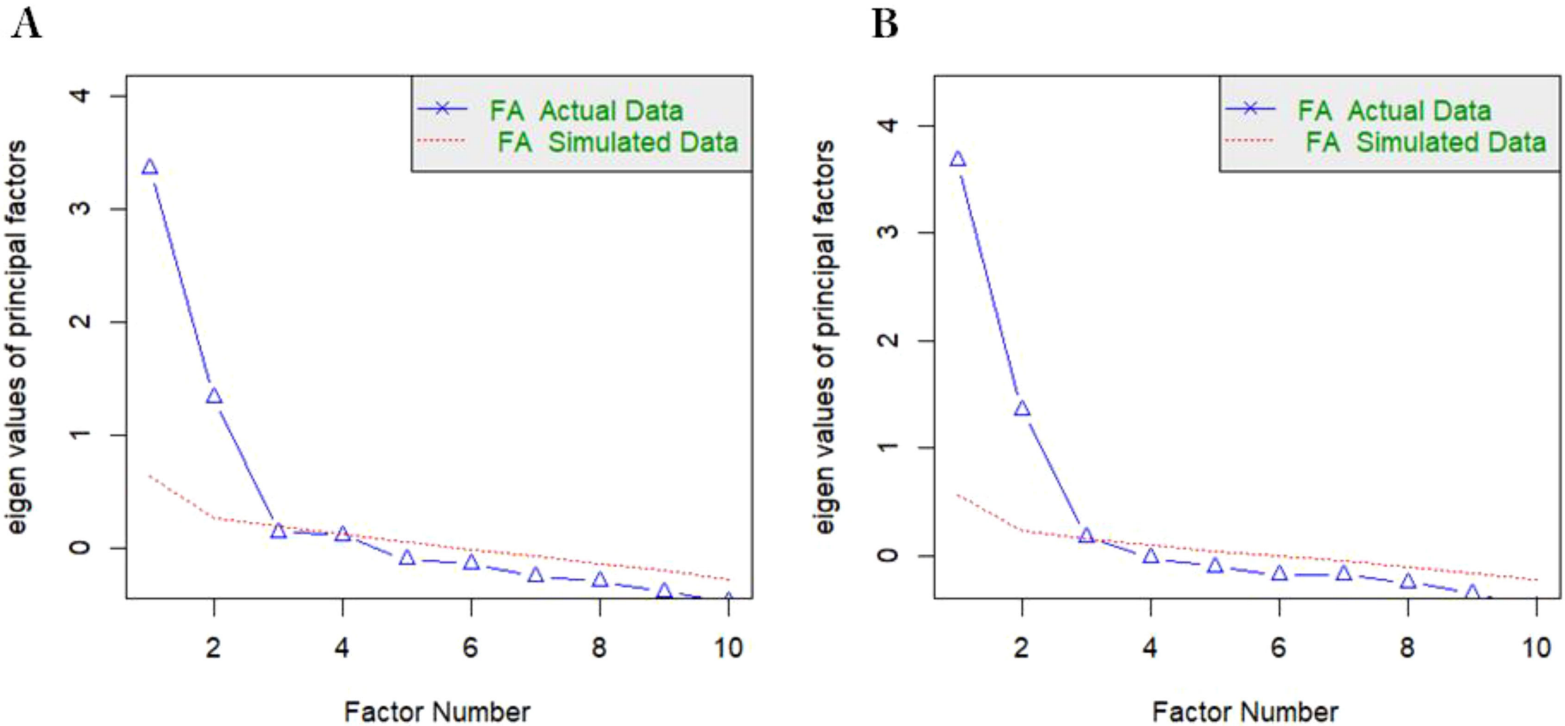
Scree plot with parallel analysis of the PSS-10 among second and third trimester pregnant woman. **(A)** second trimester, **(B)** third trimester, Parallel analysis suggests that the number of factors = 2.

**Table 2** presents a comprehensive overview of the descriptive statistics, internal consistency reliability, and the outcomes of the exploratory factor analysis (EFA) for each item of the Perceived Stress Scale (PSS-10), stratified by gestational age subgroups. The metrics of absolute skewness and kurtosis for all item scores were observed to be below the threshold of 1, indicating adherence to a normal distribution, a prerequisite for the application of parametric statistical tests. The adequacy of the sample for factor analysis was evaluated using the Kaiser-Meyer-Olkin (KMO) measure, with results indicating satisfactory values of 0.817 and 0.853 for the second and third trimester pregnancy samples, respectively. Bartlett’s test of sphericity further confirmed the suitability of the data for exploratory factor analysis (EFA), yielding significant results (χ² = 803.81 for the second trimester and χ² = 1285.03 for the third trimester; both p < 0.001). These findings confirm that the dataset meets the prerequisites for conducting EFA. The EFA, augmented by the Varimax rotation technique, extricated two distinct factors from the data, each with an eigenvalue exceeding the benchmark of 1.0. At the same time, scree plot with parallel analysis results show that the two-factor model is the best (**Figure 1**). The two factors were named perceived helplessness and perceived self-efficacy, respectively. These factors were found to account for a substantial proportion of the variance in the sample, specifically 60.58% for the second trimester and 63.22% for the third trimester. This finding underscores the presence of two salient factors that underlie the perception of stress among pregnant women. Factor loadings, which represent the correlation between each item and its corresponding factor, were observed to range from 0.58 to 0.88 across the two groups, suggesting a moderate to strong association. These loadings provide evidence of the construct validity of the PSS-10 items in relation to the perceived stress factors.

**Table 2:** Internal consistency reliability and exploratory factor analysis of the PSS-10 among second and third trimester pregnant woman.

Short item names	Second trimester (n=201)			Third trimester (n=293)		
	Mean ± SD	Factors		Mean ± SD	Factors	
		Perceived helplessness	Perceived self-efficacy		Perceived helplessness	Perceived self-efficacy
1. Been upset	1.90 ± 0.75	0.702		1.85 ± 0.77	0.767	
2. Unable to control	1.64 ± 0.74	0.820		1.74 ± 0.76	0.843	
3. Nervous and stressed	1.61 ± 0.73	0.815		1.68 ± 0.78	0.866	
6. Could not cope	1.91 ± 0.82	0.677		1.92 ± 0.86	0.581	
9. Been angered	1.81 ± 0.85	0.669		1.93 ± 0.92	0.689	
10. Could not overcome	1.73 ± 0.74	0.716		1.75 ± 0.81	0.727	
4. Felt confident	3.36 ± 1.20		0.784	3.50 ± 1.25		0.737
5. Going your way	3.21 ± 1.12		0.804	3.45 ± 1.23		0.825
7. Control irritations	3.56 ± 1.09		0.848	3.71 ± 1.11		0.877
8. On top of things	4.01 ± 1.04		0.749	3.98 ± 1.07		0.768
Eigenvalue		4.012	2.046		4.279	2.043
Variance percent (%)		40.119	20.463		42.786	20.432
Total variance (%)		60.583			63.219	

### 3.3 Reliability

The reliability analysis demonstrated strong internal consistency for the PSS-10 in both trimester groups. For second-trimester pregnant women, the Cronbach’s α coefficients were 0.838 for the perceived helplessness subscale and 0.824 for the perceived self-efficacy subscale, with an overall Cronbach’s α of 0.823. McDonald’s omega coefficients for perceived helplessness and perceived self-efficacy were 0.842 and 0.823, respectively. Among third-trimester pregnant women, slightly higher Cronbach’s α values were observed, with 0.860 for perceived helplessness and 0.834 for perceived self-efficacy, yielding an overall Cronbach’s α of 0.840. Correspondingly, McDonald’s omega coefficients were 0.863 for perceived helplessness and 0.835 for perceived self-efficacy, further affirming the scale’s reliability.

### 3.4 Confirmatory factor analysis

**Figure 2:**
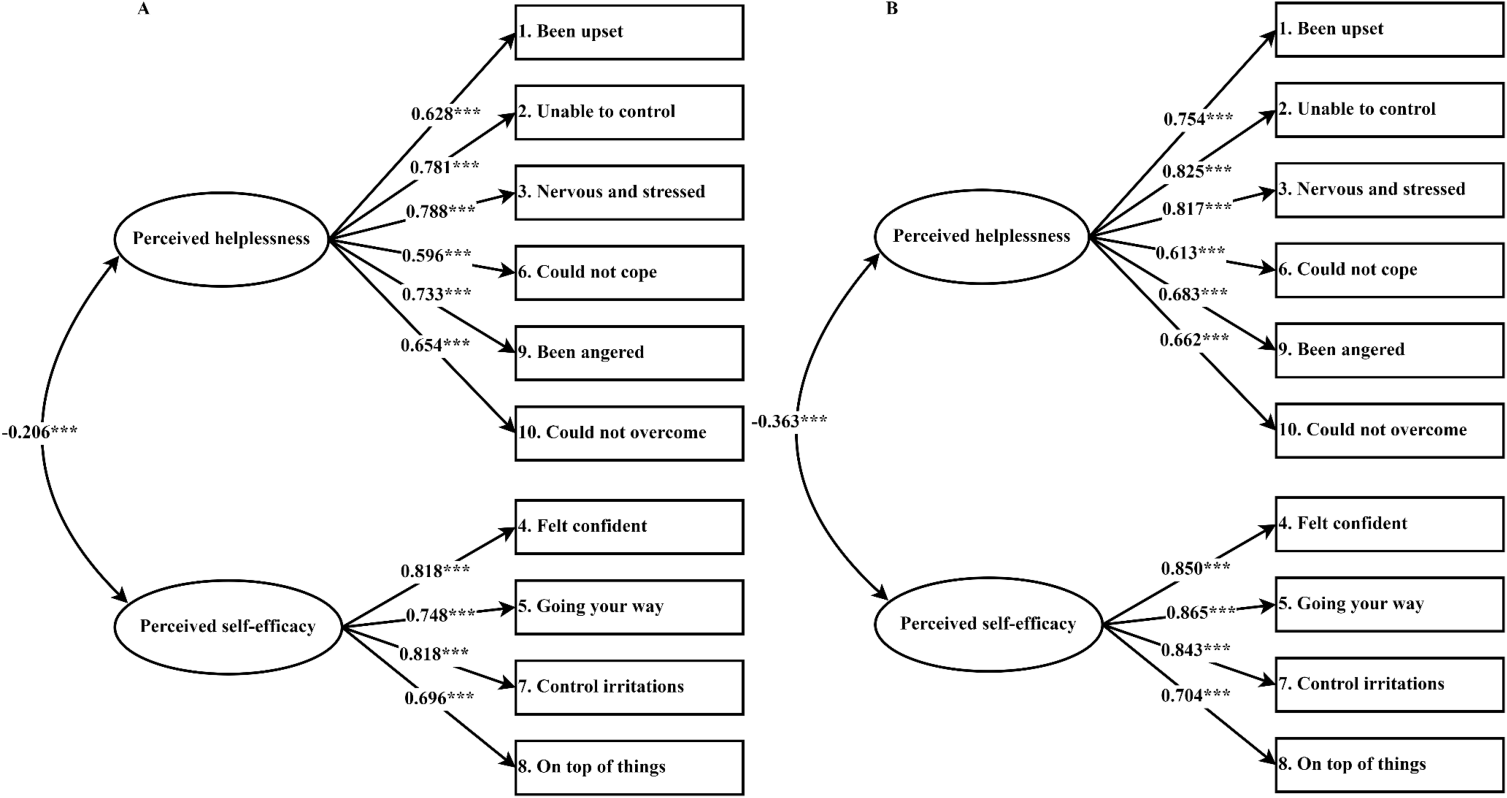
Standardized factor loadings for the two-factor structure model of the PSS-10 among second and third trimester pregnant woman. **(A)** second trimester, **(B)** third trimester, *** indicates that P-valueless than 0.001.

The confirmatory factor analysis (CFA) was conducted to scrutinize the factor structure of the Perceived Stress Scale (PSS-10) among pregnant women in their second and third trimesters. The model fit indices derived from the individual CFAs for each group are delineated in **Table 3**. The Sattora-Bentler scaled χ² statistic, a stringent test of model fit, indicated significance at the 1% level for both trimester groups, ostensibly suggesting a poor model fit. However, acknowledging the heightened sensitivity of the χ² statistic to sample size, particularly in large samples such as those under investigation, the assessment was supplemented by a suite of alternative fit indices. For both the second and third trimester groups, the Standardized Root Mean Square Residual (SRMR) and the Root Mean Square Error of Approximation (RMSEA) yielded values below the threshold of 0.08. Concurrently, the Tucker-Lewis Index (TLI), Goodness of Fit Index (GFI) and Comparative Fit Index (CFI) surpassed the benchmark of 0.90, reinforcing the model’s adequacy. In pursuit of establishing measurement invariance across the two sub-groups, robust maximum likelihood estimation was engaged to evaluate a hierarchy of invariance models. The results demonstrated that all indices adhered to the established criteria, thereby supporting the invariance of the measurement model. As illustrated in **Figure 2**, the factor loadings for the PSS-10 items exhibited a moderate to strong range, from 0.60 to 0.82 for the second trimester samples and from 0.61 to 0.87 for the third trimester samples. These loadings, indicative of the degree to which each item defines its respective factor, collectively substantiate the two-factor structure of the PSS-10 as applicable to both second and third trimester pregnant women.

**Table 3:** Goodness-of-fit indices of individual & multigroup confirmatory factor analyses.

	χ2	df	p-value	CMIN/DF	GFI	CFI	TLI	SRMR	RMSEA (90%CI)
Second trimester (n=202)	106.295	34	<0.001	3.126	0.946	0.952	0.923	0.068	0.065 (0.057, 0.073)
Third trimester (n=294)	115.880	34	<0.001	3.408	0.942	0.960	0.922	0.073	0.069 (0.060, 0.078)
Configural	282.16	68	<0.001	4.149	0.949	0.958	0.921	0.064	0.063 (0.055, 0.071)
Metric	292.72	76	<0.001	3.852	0.946	0.957	0.923	0.069	0.062 (0.052, 0.072)
Scalar	296.32	79	<0.001	3.751	0.946	0.956	0.924	0.067	0.061 (0.052, 0.070)
Strict	323.38	89	<0.001	3.634	0.942	0.954	0.923	0.068	0.061 (0.053, 0.069)

df, degrees of freedom; DMIN/DF, ratio of chi-square value to degrees of freedom; GFI, goodness-of-fit index; AGFI, adjusted goodness-of-fit index; CFI, comparative fit index; SRMR, standardized root mean square residual; RMSEA, root mean square error of approximation.

### 3.5 Concurrent validity evidence based on relationships with other variables

**Table 4** presents a detailed exposition of the relationships between Perceived Stress Scale (PSS-10) scores and corresponding measures designed to assess concurrent validity. Among the second-trimester pregnant women, the total PSS-10 scores manifested moderate positive correlations with CMHS constructs (r= 0.40 to 0.54). Factor 1 (perceived helplessness) demonstrated strong positive correlations with CMHS constructs (r= 0.59 to 0.72). In contrast, Factor 2 (perceived self-efficacy) demonstrated small negative correlations with CMHS constructs (r= -0.10 to -0.19). Parallel patterns were discerned within the third-trimester pregnant women: the total PSS-10 scores evinced moderate positive correlations with CMHS constructs (r= 0.38 to 0.57). Factor 1 displayed strong positive correlations with other CMHS constructs (r= 0.56 to 0.72), and Factor 2 demonstrated a small negative correlation with CMHS constructs (r= -0.11 to -0.24). These collective findings corroborate the PSS-10’s reliability as an instrument for the assessment of perceived stress among pregnant women across varying stages of gestation.

**Table 4:** Correlations of PSS-10 and its two subscales with CMHS constructs among second and third trimester pregnant woman.

Variables	Second trimester (n=403)			Third trimester (n=587)		
	Perceived helplessness	Perceived self-efficacy	PSS-10	Perceived helplessness	Perceived self-efficacy	PSS-10
Interpersonal sensitivity	0.713^***^	-0.127^*^	0.512^***^	0.675^***^	-0.168^**^	0.503^***^
Poor endurance	0.703^***^	-0.154^**^	0.523^***^	0.718^***^	-0.230^***^	0.566^***^
Poor adaptability	0.701^***^	-0.180^**^	0.538^***^	0.692^***^	-0.224^***^	0.547^***^
Unbalanced mind	0.654^***^	-0.127^*^	0.476^***^	0.561^***^	-0.172^**^	0.378^***^
Emotion disorder	0.642^***^	-0.179^**^	0.439^***^	0.688^***^	-0.235^***^	0.551^***^
Anxiety	0.716^***^	-0.101^*^	0.497^***^	0.681^***^	-0.138^**^	0.490^***^
Depression	0.701^***^	-0.129^**^	0.506^***^	0.662^***^	-0.114^**^	0.463^***^
Hostility	0.593^***^	-0.171^**^	0.404^***^	0.639^***^	-0.168^**^	0.482^***^
Paranoid ideation	0.628^***^	-0.191^***^	0.502^***^	0.638^***^	-0.230^***^	0.518^***^
Somatization	0.605^***^	-0.109^*^	0.435^***^	0.666^***^	-0.193^***^	0.513^***^

^*^P<0.05, ^**^P<0.01, ^***^P<0.001.

## 4 Discussion

This investigation represents the inaugural effort to appraise the psychometric attributes of the Chinese version of the Perceived Stress Scale (PSS-10) within a substantial cohort of 990 pregnant women, stratified across the second and third trimesters of gestation. The present study’s contribution to the literature is underscored by its pioneering role in evaluating the scale’s psychometric integrity within this demographic context in China. The sequential application of exploratory factor analysis (EFA) and confirmatory factor analysis (CFA) yielded evidence of the PSS-10’s commendable construct validity and internal consistency reliability among the Chinese pregnant women sampled. The Cronbach’s α coefficients, which surpassed the threshold of 0.70 for both total and subscale scores, attested to the PSS-10’s internal consistency reliability. And McDonald’s omega coefficients surpassed the threshold of 0.70, further affirming the scale’s reliability. Overall, our study suggests that PSS-10 is suitable for measuring perceived stress among Chinese pregnant women.

Our study supports a two-factor structure of PSS-10 terms, which has been confirmed by most previous studies (21, 22, 41–43). As expected, our study of the second and third trimesters yielded similar results, with the two principal factors representing negative and positive emotions, respectively. We found that the model was well replicated. The variance obtained in this study (60.58% and 63.22% in pregnant women in the second and third trimesters, respectively) was acceptable and ranged from 40% to 60% as usual in sociological studies (44, 45). In addition, in this study, factor loads ranged from 0.58 to 0.88 for all items, with all items loading more than 0.5 for one of the factors, this indicates that all PSS -10 programs contribute significantly to the measurement of perceived stress in Chinese pregnant women.

The Cronbach’s α and McDonald’s omega value of the PSS-10 in our study with the Chinese pregnant women population demonstrates strong internal reliability, echoing the findings from similar studies conducted in Brazil (46)and the United States (47). The uniformity in Cronbach’s α and McDonald’s omega across these diverse populations underscores the universal applicability of the PSS-10. On one hand, it suggests that the scale is robust enough to measure psychological stress in different cultural contexts, providing a reliable metric for comparative studies. On the other hand, the cross-cultural reliability of the PSS-10 has significant implications for global health research, particularly in understanding and addressing the mental health needs of pregnant women worldwide. It enables the development of informed, culturally-sensitive interventions. However, it is also crucial to consider potential challenges in cross-cultural research. These include language translation, idiomatic expressions, and cultural interpretation of stress indicators, which may require additional qualitative research to complement quantitative findings (48).

Structural validity refers to the extent to which a set of measured variables reflects the theoretical potential structure that these indicators should measure. Estimating the correlation between different structures is the key to solving the structural validity. The construct validity was examined based on factor loading. Ideally, all factor loads should be greater than 0.50 and statistically significant. Our CFA results found that all factor loads were ≥0.50 for both sample populations, a finding that is consistent with measurements in Chinese community samples (27). In addition, the model performed well in many fitting indexes except chi-square test (χ2). As we all know, chi-square is greatly affected by the sample size. When the sample size is more than 200, it cannot be used as a fitting evaluation index of CFA (49). Moreover, the two-factor PSS-10 model of the two sample populations showed a good fit index (RMSEA < 0.08; TLI, CFI and GFI > 0.90), indicating that the PSS-10 model showed sufficient fitness in the Chinese pregnant women population. In addition, we evaluated measurement invariance, and the two-factor model was strictly invariant between the second-trimester sample and the third-trimester sample, illustrating that PSS-10 was universally applicable in both sample populations.

In the domain of concurrent validity, our analyses revealed significant associations between the PSS-10 factors and the CMHS constructs. Notably, the anxiety and stress dimensions of the CMHS exhibited the highest degree of correlation with the negative emotion subscale of the PSS-10, attributed to the conceptual alignment and structural similarity between these constructs. This strong correlation underscores the theoretical and empirical interplay between perceived stress and anxiety- or stress-related mental health constructs (50, 51). The collective results of these validity assessments substantiate the PSS-10 as a reliable and valid instrument for quantifying perceived stress among pregnant women, irrespective of their gestational stage. These findings contribute to the validation of the PSS-10 in the context of Chinese pregnant populations and support its application in psychological research and clinical practice.

There are also some limitations to this study. First, the pregnant women in this study were recruited from a municipal maternal and child health center, so our sample may not be representative of all pregnant women. Second, since this study was conducted in an urban health maternal and child health center, it is recommended that a similar study be carried out in rural populations, with caution in its dissemination. Third, only self-reported measures were used in this study, so participants’ responses may be biased to some extent by subjective influences such as social expectations. In order to avoid this problem, it is necessary to adopt more objective behavior test in future research. Finally, while this study enrolled a substantial sample size of pregnant women across different trimesters, we were unable to compare the sociodemographic characteristics of our participants with those of the general population due to the absence of comprehensive data on pregnant women in national and provincial statistics. This limitation may impact the generalizability of our findings, particularly if the recruited sample is not fully representative of the diverse sociodemographic profiles present in the broader population. Future studies could benefit from collaborations with national health authorities to obtain more granular population-level data or expand recruitment to include a more geographically and demographically diverse sample.

## 5 Practical implications

The findings of this study highlight the utility of the PSS-10 as a reliable and culturally appropriate tool for assessing perceived stress among Chinese pregnant women, with significant implications for clinical practice and public health. Its validation supports the integration of stress screening into routine prenatal care, enabling early identification and tailored interventions to address specific stress domains such as perceived helplessness and self-efficacy. This can guide the development of culturally sensitive mental health programs that account for unique stressors in Chinese society, such as traditional beliefs and family dynamics. Additionally, the PSS-10’s robust psychometric properties across gestational stages facilitate its use in both cross-sectional and longitudinal studies, promoting a deeper understanding of stress dynamics during pregnancy. By extending its application to diverse populations, including rural areas, this tool can help reduce health disparities and improve maternal mental health outcomes, ultimately fostering healthier pregnancies and supporting the well-being of mothers and their children.

## 6 Conclusion

In summation, the findings of this study endorse the PSS-10 as a psychometrically sound instrument for quantifying perceived stress among Chinese pregnant women. The scale’s demonstrated suitability for this population underscores its potential utility in both epidemiological research and clinical assessments, facilitating a nuanced understanding of stress dynamics during pregnancy. This academic recapitulation of the study’s findings encapsulates the methodological rigor and the significance of the psychometric validation of the PSS-10 within the Chinese pregnant population, positioning the scale as a reliable tool for future investigations in this domain.

## Data Availability

The raw data supporting the conclusions of this article will be made available by the authors, without undue reservation.
